# Data Access Based on a Guide Map of the Underwater Wireless Sensor Network

**DOI:** 10.3390/s17102374

**Published:** 2017-10-17

**Authors:** Zhengxian Wei, Min Song, Guisheng Yin, Houbing Song, Hongbin Wang, Xuefei Ma, Albert M. K. Cheng

**Affiliations:** 1 College of Computer Science and Technology, Harbin Engineering University, Harbin 150001, China; weizhengxian@sina.com (Z.W.); yinguisheng@hrbeu.edu.cn (G.Y.); wanghongbin@hrbeu.edu.cn (H.W.); 2 System Engineering Innovation Center, Systems Engineering Research Institute, Beijing 100094, China; 3 Information Technology Centre, Beijing Foreign Studies University, Beijing 100089, China; songmin@bfsu.edu.cn; 4 Department of Electrical, Computer, Software, and Systems Engineering, Embry-Riddle Aeronautical University, Daytona Beach, FL 32114, USA; h.song@ieee.org; 5 College of Underwater Acoustic Engineering, Harbin Engineering University, Harbin 150001, China; 6 National Key Laboratory of Underwater Acoustic Science and Technology, Harbin Engineering University, Harbin 150001, China; 7 Department of Computer Science, University of Houston, Houston, TX 77204, USA; cheng@cs.uh.edu

**Keywords:** underwater wireless sensor networks, data access, data guide map, metadata, center ring

## Abstract

Underwater wireless sensor networks (UWSNs) represent an area of increasing research interest, as data storage, discovery, and query of UWSNs are always challenging issues. In this paper, a data access based on a guide map (DAGM) method is proposed for UWSNs. In DAGM, the metadata describes the abstracts of data content and the storage location. The center ring is composed of nodes according to the shortest average data query path in the network in order to store the metadata, and the data guide map organizes, diffuses and synchronizes the metadata in the center ring, providing the most time-saving and energy-efficient data query service for the user. For this method, firstly the data is stored in the UWSN. The storage node is determined, the data is transmitted from the sensor node (data generation source) to the storage node, and the metadata is generated for it. Then, the metadata is sent to the center ring node that is the nearest to the storage node and the data guide map organizes the metadata, diffusing and synchronizing it to the other center ring nodes. Finally, when there is query data in any user node, the data guide map will select a center ring node nearest to the user to process the query sentence, and based on the shortest transmission delay and lowest energy consumption, data transmission routing is generated according to the storage location abstract in the metadata. Hence, specific application data transmission from the storage node to the user is completed. The simulation results demonstrate that DAGM has advantages with respect to data access time and network energy consumption.

## 1. Introduction

In the 21st century, there has been increasing concern with respect to the development of the world economy and the military, and the ocean has become an important base for humans to maintain their survival and the sustainable development of society. Worldwide, there has been more and more attention given to maritime rights and interests. There has therefore been a boom in global, modern ocean technology research, specifically with respect to marine resource exploitation and ocean energy utilization, representing a new technological revolution across the globe [1,2]. With the rapid development of sensor and wireless network technologies, underwater wireless sensor networks (UWSNs) have gradually become the focus of new research interest because of their low cost, high reliability, and potential for a wide range of applications.

When compared to wireless sensor networks based on radio communication, UWSNs are very different in terms of network composition, deployment and networking patterns. This is because of differences in utilization objectives, the environment, the scope of work and the patterns of communication. Among them, UWSNs used for deep ocean marine exploration and monitoring are composed of floating or submerged floating sensor nodes, mobile nodes (Unmanned Underwater Vehicles or UUVs and Autonomous Underwater Vehicles or AUVs) and observation nodes (ships), to form a network system. The sensor nodes carry out real-time monitoring and collect a variety of data distributed in the monitoring region. By way of underwater acoustic communication [3] or up-water radio communication, they send the specific application data (such as target detection information and ocean environmental information) to the mobile node or observation node. The mobile nodes (UUVs, AUVs) can carry sensor nodes, which have the function of redeploying the sensor node and reconfiguring the network, as well as monitoring, communication, data collection, and processing. The observation node is responsible for deployment of sensor nodes, configuring the network, and collecting and processing the application data distributed in the network effectively as well.

For UWSNs, in the process of monitoring ocean environment and detecting underwater targets, first of all sensor nodes are distributed in a wide range for underwater monitoring and collection of the environmental information and target information. Then, this is stored by an appropriate mechanism. For the collection and processing of the distributed data, it can be divided into two types: non-real-time data (such as environmental information and data) and real-time data (such as military target and rescue target information). At the same time, in a specific scenario such as search and detection of underwater targets for rescue, the observation node should reduce the times and distances of active communication as far as possible on collecting the distributed data in the network. This is in order to lower the background noise in the UWSN. Underwater acoustic communication is mainly used in UWSNs, and is characterized by low bandwidth, high delay and higher bit error rate, showing great differences on comparison with sensor networks based on radio communication from the perspective of transmission reliability, transmission delay, energy consumption on storage, and query data. The above has brought about new challenges for UWSN data storage, discovery and query [4,5,6]. The main challenges are as follows:

(1) Efficient UWSN data access includes storage, discovery and query. Networks of different types generate different types of data, and the data on the sensor node needs to be collected and stored efficiently. At the same time, the query sentences from the users need to be dealt with as far as possible in order to return different granularity data (especially in military application and emergency rescue) to the users with the smallest delay possible.
(2) There is the problem of energy consumption in the process of data storage and query. Energy consumption is huge due to the long distances and long underwater data transmission times, and this will shorten the network service life while increasing background noise in UWSN. Hence, in the processing of data storage and query, energy consumption of data transmission needs to be reduced and balanced as much as possible for the purpose of prolonging the network lifetime.

To address the challenges mentioned above, the goal of our work is to provide data access mechanism for storage and query real time data with less query time and energy consumption as possible. At the same time, this mechanism can support data discovery and large volume data aggregation for UWSNs. For this aim, a data access based on a guide map (DAGM) for the underwater wireless sensor networks was proposed in this paper, which is based on the metadata [7] and ring structure [8]. In DAGM, according to the shortest average user data query path of the network, a center ring structure composed of nodes was established for storing the metadata, which describes the abstracts of data content and storage location. For prolonging the network lifetime, the scope of network is divided into different areas. The sensor nodes in an area are formed into a cluster. When data is generated in the sensor node, the storage node is determined by the geographic harsh table (GHT) [9,10]. The data is transmitted from the sensor node (data generation source) to the storage node being stored and the metadata generated for it, and metadata is sent to the center ring node that is the nearest to the storage node. After that, metadata is diffused and synchronized at the other nodes within the center ring, so every center ring node can store all metadata in a set for the network, and the data map can be formed. In the process of data query, when there are query data in any node (observation nodes, also called sink nodes are query users generally), the center ring node that is the nearest to it should be selected to deal with the query sentence. If there is no data meeting the user requirements in the map data, the query result “*query failed*” is given to the user; if there is existing data that the user requires, data transmission routing will be generated according to the storage location abstract described by the metadata, and the routing and specific data requests will be sent to the storage node. At the same time, the query result “*ready to receive data*” and data transmission routing will be returned to the query user. Lastly, specific application data transmission could be completed by the user and the storage node according to data transmission routing.

In our study the main contributions are as follows:

(1) The DAGM system model is proposed so that the user (observation node) in an UWSN can access the specific application data and events it is concerned with as much as possible. The most challenging and difficult problems of DAGM have been analyzed;
(2) The framework of DAGM is established. According to the shortest average query path of the network, the center ring structure composed of nodes is established for storing the metadata, and then the data map of the network is formed. In the meantime, data storage and query mechanisms are established to aid user access-specific application data and events quickly;
(3) Data storage delay, data query delay, and a network energy consumption calculation model of DAGM are established, providing a method for the analysis of DAGM performance.

The second section gives the system model and problem description of the work in this paper. The construction of data storage and query, architecture of the data map, and the mode of data storage and query will be discussed in Section 3. In Section 4, the performance of DAGM with respect to data access and energy consumption is discussed in detail. Simulation experiments and their results are given in Section 5. In Section 6, we provide recent research advances in the data storage and query of the wireless sensor network. The final section presents the conclusions of the paper.

## 2. Related Works

Nowadays, as new applications of wireless sensor network need data processing with temporal constraints in their tasks, one of the new challenges for wireless sensor networks is the handling of data access [11,12]. There are three research aspects in wireless sensor network data access: data storage [13,14,15,16], data aggregation [17,18,19,20] and data query [21,22,23,24]. These prior studies have looked at maximizing the energy efficiency in order to increase the lifetime of wireless sensor network, or focus on the time performance on data access of the wireless sensor network from a single aspect. As we acknowledge, the energy efficiency and time performance of wireless sensor networks are the result of a compromise between the data storage schemes, data aggregation methods, and data query strategies. Our study systematically investigates the data storage schemes and data query strategies through balancing the performance between access time and energy consumption of the underwater wireless sensor network.

Data storage is the first step in data processing in the wireless sensor network. According to the different data storage strategies, storage can be divided into centralized storage, local storage, and distributed storage [9,25,26]. As for the analytical modeling of storage schemes and query strategies to increase the lifetime of the network and decrease time delays with respect to data access, there are two interesting categories studied: tree-based and grid-based protocols. The tree-based protocols include the method shown by Saxena of [27], as well as SDS [25], Scoop [28], DISAGREE [29], and so on. Grid-based protocols include the schemes shown by the authors of [14], [30,31] and [16], etc. 

However, these studies did not consider real-time events and non-real-time data existing in the network simultaneously for data storage and querying. We focus on the access mechanisms for emergency real-time events and data acquisitions. Furthermore, our schemes can be used in non-real-time data acquisition with attractive time performance and energy consumption in the wireless sensor network.

As we know, when comparing underwater acoustics and radio, there are great differences in communication bandwidth, delay and bit error rate. Hence, data storage schemes and query strategies of wireless sensor networks based on radio communication cannot be used in UWSNs directly [32,33]. Recently, many methods for data storage, data aggregation and collection, data query, and acquisition in UWSNs have been proposed [20,21,22,34,35,36,37,38,39,40]. For the data query, [21] proposed a sub-region query processing mechanism called QPM. In QPM, a routing tree is constructed where a sensor node corresponds to a tree node, and sensor node acknowledges whether it lies within the sub-region of interest or not, therefore it can complement the querying data function in the sub-region. For the data and event discovery and acquisition, work [36] provided a mechanism to gather and route sensory data to a sink node (SN) in order to detect events and determine event sources. For SDA, when an event occurs, sensory data of multiple neighboring sensor nodes are gathered by a sensor node (called a relay node) and are aggregated into a data packet for routing to a SN. Data storage, data discovery and aggregation, data query and acquisition of wireless sensor networks must be based on suitable network routing protocols [27,41]. Many researchers have focused on this theme. Reference [42] presented a routing algorithm named Prolong-SEP (P-SEP). Reference [43] proposed an efficient routing algorithm for preserving k-coverage and the reliability of data with logical fault tolerance. Otherwise, for the wireless sensor networks or underwater wireless sensor networks, some researchers have focused on distributed database management [44], data gathering [45,46], etc.

These methods are realized through the geographic hash functions or other mechanisms to complete data storage, and show good performance advantages with respect to data storage, query and acquisition in special backgrounds. However, the disadvantage is that the data query does not achieve the shortest average query path. 

## 3. System Model and Problem Description

### 3.1. System Model

**Figure 1:**
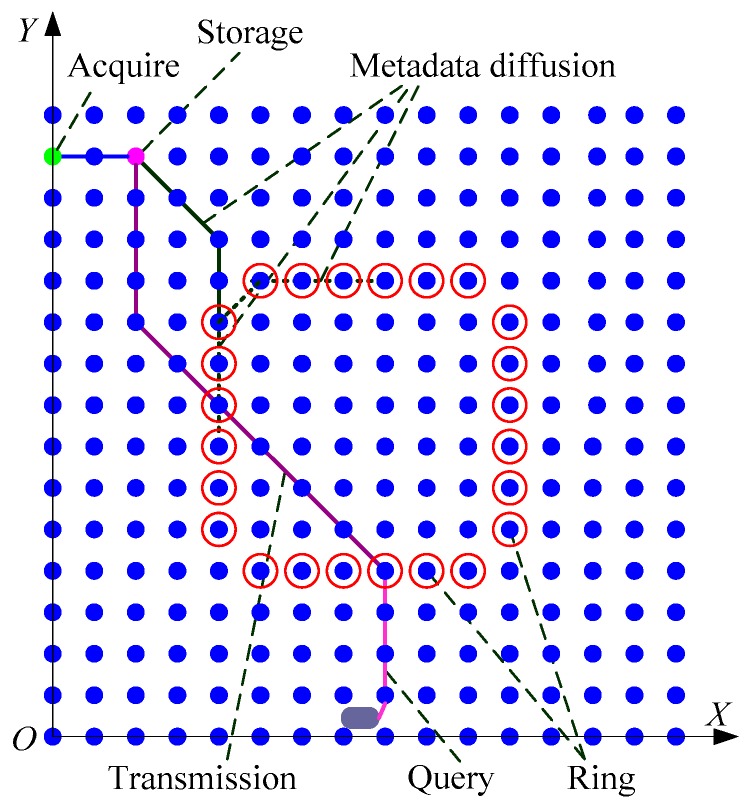
Data access based on a guide map (DAGM) mechanism.

We assume *n* × *n* sensor nodes are uniform or randomly distributed in a square area where the length of a side is *L*, as shown in Figure 1. The observation node is the data query user, randomly located within the network, and it can query different granularity and quality data in accordance with certain query conditions.

In this paper, the square area of the UWSN does not limit the model’s generalization, because when a network adopts a circle shape region, the circumscribed square of this circle can be considered as the network region, and when the sensor nodes are randomly deployed, according our previous work [42], every sensor node can be can be considered to deploying in regular square lattice that the network region is divided regularly. Therefore, the model and method of this paper can be applied to other network models, and the specific assumptions are as follows:

(1) The nodes are divided into three types: sensor nodes, storage nodes and center ring nodes. Each node can obtain its own location information (through the global positioning system (GPS) or locating protocol). The network is connected, and any two nodes can communicate through the multiple hops, assuming that the node has a small mobile range but it does not affect the communication and undermine the connectivity of network.
(2) The network works in a two-dimensional plane. The nodes are deployed according to square lattice, with the network scope divided into regular square lattice. Each lattice has an ID number, and in terms of the nodes located in it, when each lattice consists of multiple sensor nodes, only one node is working, while the others are sleeping. Each node knows the situation of the deployment, such as the scope of network, neighbor nodes, and so on.
(3) Each node can be considered as a data query user. They can send a data query sentence to the nearest center ring node at any time, and will not leave the location out of the communication range before the end of data query.

Based on the above assumptions, the basic ideas of DAGM are:

(1) To establish the center ring structure. In the process of deployment, the scope of network is well divided into *n* × *n* square lattices, and sensor nodes are deployed at the inside of the square lattice. Then, the center ring structure is established as composed by nodes according to the shortest average user query path in the network for storing the metadata.
(2) To store data in the network. When the sensor nodes generate data by monitoring the environment and detecting the target, the lattice ID number and location of the storage node is determined by the geographic harsh table (GHT). At the same time, the data will be transmitted to the storage node by multiple hops. Then, after the data stored, the storage node generates metadata that include the content abstract and storage location abstract for it.
(3) To establish a data map for the network. When there is metadata generated in any of the storage nodes, it will be sent to a center ring node which is the nearest from it. This center ring node will store the metadata and become a metadata set. At the same time, metadata will be diffused and synchronized to other center ring nodes in a double direction, so that the data map of the network will be formed because every center ring node has stored the metadata of the network.
(4) To query data from center ring node that is the nearest from it. When each node queries data, it will send the query sentence to the nearest center ring node from it. After the center ring node receives the query sentence, it will try to find whether data is exiting the network from the data content abstract that is stored in map data. If the data does not satisfy the query sentence, “*query failed*” is informed to the user. If there is data that the user requires, data transmission routing will be generated according to the storage location abstract described by the metadata. After that, the routing and specific data requests will be sent to the storage node. At the same time, the notification of query result “*ready to receive data*” and data transmission routing will be returned to the user.
(5) To transmit the specific application data. After the user receives the notification “*ready to receive data*”, the data is transmitted according to the data transmission routing, which is completed together with the sensor node and storage node.

For the basic ideas of DAGM, it cannot only carry the storage and query real time data with less query time and energy consumption as possible, but also support data discovery, large volume data aggregation and a lot of data requests from one user for UWSNs. The data storage, metadata diffusion, data query and data transmission of DAGM are as shown in Figure 1.

In Figure 1, *n* × *n* sensor nodes are distributed in the square area where the length of a side is *L*. Each node occupies a square lattice and the length of lattice is *g*. The relationship between nodes distance *R*, communication radius *r_b_* and detection radius *d_b_* is *r_b_* > 2*d_b_*, *r_b_* > $\sqrt{2}R$, and two neighbor nodes in the network can communicate through a single hop. We built the coordinate system for the sensor node-deployed plane; the node in the lower left corner is the origin, and its coordinate is (*X*_0_, *Y*_0_). According to the deployment of sensor nodes, each node corresponds to a square lattice, and each square lattice has an ID number *G*(*G_X_*, *G_Y_*). Then, we can obtain the formula *G_X_* = $\left\lfloor \frac{X-X_{0}}{g}\right\rfloor$, *G_Y_* = $\left\lfloor \frac{y-y_{0}}{g}\right\rfloor$.

Assuming that each node has a global information table *Tab* (*ID*, *C*, *G*, *Ad_j_*) of the network, *ID* represents the node identification number; *C* represents the node coordinate; *G* represents the node lattice ID number; and *Ad_j_* represents the neighbor nodes. Because the sensor node has a limited communication radius, it must know which nodes are neighbor nodes and can communicate with it in a single hop. At the initialization of the network, each sensor node will broadcast its own *ID* in a single hop, and obtain the node *ID* that other nodes published. Thus, each node can know the neighbor nodes they can communicate with. In addition, in the global information table, the *Ad_j_* of the corresponding record is set to 1. After that, the node sends a message to the neighbor nodes in every certain period, and informs them of the update condition (nodes join in or get out) in the global information table they stored. After receiving the update message from neighbor nodes, the local global information table in the nodes is updated. In each node, if the message from a neighbor node was not received in a predetermined period, that means this neighbor node is invalid, and the corresponding record is deleted in the local global information table.

### 3.2. Problem Description

An important purpose of UWSN is to make the user (such as the observation node) obtain the required data as fast as possible, and to prolong the lifetime of the network. In DAGM, there are three stages from the sensor nodes generating data to the user getting data, that are: generation and storage of data, diffusion and synchronization of metadata, and query and receipt of data. In DAGM, to make the user query and receive data of different granularity and quality as fast as possible, at first, when sensor nodes generate data, the UWSN will not only store data, but it will also generate metadata and diffuse it to the center ring. Secondly, in the user query data, the UWSN not only can send the query sentence to the nearest center ring node, but it can also transmit the data with a data transmission routing strategy. This can not only reduce the delay in data access, but also save network energy and prolong the network lifetime. When it comes to the time performance of network, it contains two parts. One part is the time of data storage and the metadata diffusion, and the other is the interval from user sent out the data query sentences to who receives the specific application data. Similarly, the network energy consumption also includes two parts. One is the energy consumption of data storage and metadata diffusion, and the other is energy consumption of the data query and specific application data transmission.

The data is generated after the sensor nodes monitor the environment or detect the target, and then it is transmitted to the storage node and the metadata generated for it. Then, metadata is sent to center ring node that is the nearest to the storage node and is diffused and synchronized at the other nodes within the center ring, so the data map be formed. It is only in this period where the user cannot find the data in the network when it sends out the query sentence to the network. However, the probability is very low. Not considering this case, one important problem of UWSN is ensuring a minimum time interval from the user sending a data query sentence to it receiving specific application data. Another important problem is ensuring the network energy consumption is as little as possible. Due to the communication energy consumption for data transmission being much higher than the energy consumption for data processing into the node, the time of data processing in node is much less than the time of data transmission. Hence, the delay in data query mainly includes the delay in the query sentence and specific application data transmission. At the same time, the total energy consumption of the network mainly includes energy consumption on transmission of data storage, diffusion and synchronization of metadata, as well as query sentence and specific application data.

Based on the above analysis, the core problem of the DAGM is to determine the location of the center ring nodes based on the minimum delay from the sent query sentence to received specific application data by the user, and minimizing the energy consumption of the network. The data transmission in the network is completed through multiple hops. Assuming the number of hops on sending query sentence and on querying results returning is same in each node, *k* denotes the center ring node; *i* presents other nodes exception the center ring node; and *D*(*k*, *i*) shows the number of hops from node *i* to the center ring node *k* that is the nearest from *i.* Thus, the distance sum (the hops sum, that is, the path length) from all nodes to their nearest center ring nodes is, *D*(*k*) = $\sum_{i=1}^{n\times n-m}$ *D*(*k*,*i*) , where *m* represents the number of center ring nodes.

Therefore, the center ring node k is selected from the nodes with the minimum distance sum (the shortest path) among all nodes t. Thus, we can obtain Equation (1):

(1) $$\left\{K|D(K)=\min_{1\leq t\leq n\times n}\{D(t)\}\&K\in \{1,2,\ldots ,n\times n\}\right\}$$

The center ring nodes *k* are determined by the data storage node location, user location and so on. From the basic framework of DAGM, we will analyze the building process of the center ring structure and data map, as well as methods of data storage, metadata diffusion and synchronization, and data query and specific application data transmission. 

## 4. The DAGM Framework and Method of UWSN Data Storage and Query

### 4.1. DAGM Framework and Flow

**Figure 2:**
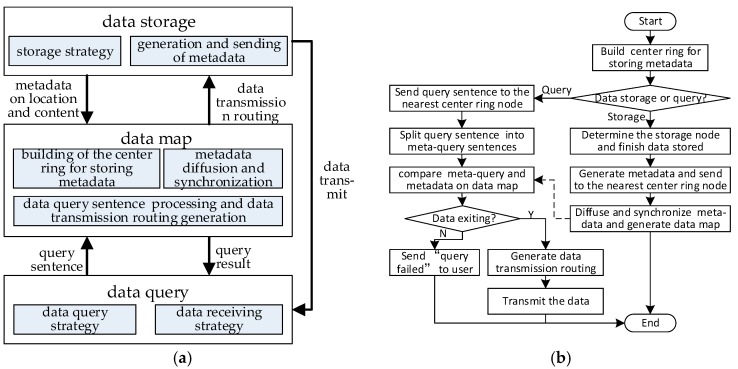
The framework and flow of DAGM. (**a**) The framework; (**b**) The flow.

In order to decrease the delay of data access and the total energy consumption in UWSN, the framework and flow of the DAGM is shown in Figure 2. The framework is divided into three parts, that are data storage, data map and data query.

Figure 2a shows the data map that is the core of DAGM. The data map includes building the center ring for storing metadata, for metadata diffusion, and for synchronization, as well as data query sentence processing, data transmission routing generation, and so on.

The procedure of DAGM is shown in Figure 2b. According the basic ideas of DAGM, firstly, the center ring that is composed of different nodes is established. When any node in the center ring receives a piece of metadata, they will merge it into the metadata set that has been stored in this node, and diffuse the metadata to the other node in the center ring along double directions. When all nodes in center ring perform same process, the metadata synchronization is finished. Thus, the data map is formed. When a center ring node receives a data query sentence, the query sentence is split into meta-query sentences. Then, the meta-query will be compared with the metadata in this node. If data is found in the network, the center ring nodes will establish data transmission routing, and return the query result “*ready to receive data*” and data transmission routing to user. At the same time, the routing and specific data requests will be sent to the storage node. If there is no data that meeting the requirement in the network, the result “*query failed*” is returned to the user.

Due to the limited energy in the network, and considering that generally sensor nodes work in clusters, we can regard one cluster as a partial area of the network. In the process of data storage, when the arbitrary sensor node *i* in an area generates data, the storage node identification number and lattice ID number of this area can be determined by GHT. Then the greedy perimeter stateless routing (GPSR) [47] is adopted to transmit data form sensor node to storage node for storing; at the same time, metadata consisting of content abstract and a storage location abstract could be generated by the storage node. After that, storage nodes will send metadata to the nearest center ring node by multiple hops.

Data query includes a query strategy and a data-receiving strategy. Under the different times and in different application backgrounds, users require different data granularity and quality of service (QoS) in the network. Sometimes they require original precise data in network, while at times they only require integration data, with types including average, minimum, maximum, and so on. Therefore, the data query sentence includes data types, granularity, QoS, etc., which can decrease redundant data that reduce the delay and energy consumption on communication. In remote data transmission of underwater environment, where the energy consumption is huge, bandwidth is low and error rate is high, so data is transmitted in network to the user by multiple hops according to data transmission routing.

### 4.2. Building Method and Processing Mechanism of the Data Map

#### 4.2.1. Building Method of the Center Ring for Metadata

In order to prolong the lifetime of the network and to reduce the time the user needs to obtain the data, center ring nodes are selected by balance and optimization between data query time and the energy consumption.

In the process of data query, every sensor node can be considered as a user, so the user is randomly located in the internal network. Therefore, the question of user query data is transformed into any node in the internal network querying data to center ring node. The nodes in the network always query data from their nearest center ring node.

Delays in data query can be divided into two parts: communication delay *Tra_t* and processing delay (that is, the delay of store-and-forward intermediate nodes) *Pro_t*. The communication delay *Tra_t* mainly depends on the communication distance and the times of awaiting retransmission. If network connectivity is confirmed, the time for awaiting retransmission is a stable value. Therefore, the question of minimum delay of any position querying data in the network can be transferred as the question that calculates the minimum hops sum of communication (or the minimum distance sum of communication) from arbitrary nodes to the nearest center ring node in the network.

**Figure 3:**
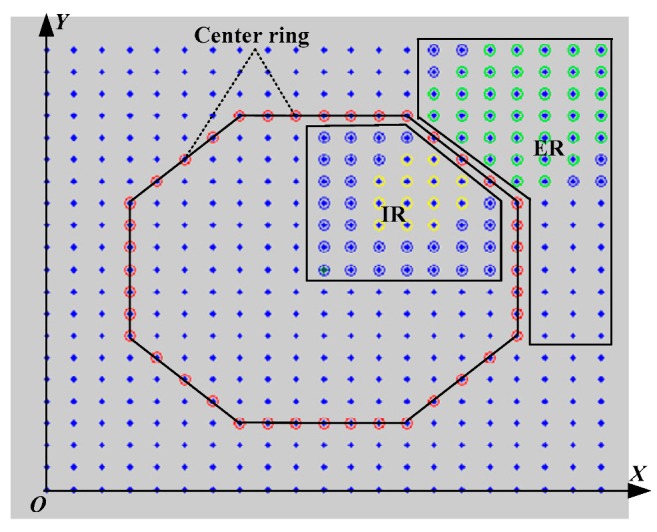
The sketch map of the center ring. IR: internal area; ER: external area.

The sensor nodes in the network are divided into the inner part and outer part by the center ring, which is shown in Figure 3. In order to calculate conveniently, we divide the UWSN into two parts in accordance with the location center ring, which include the internal area IR and external area ER. The node in the lower left corner is seen as the origin and the rectangular coordinate system is established. If distance between two nodes is a scale of the coordinate (that the coordinate value is the number of sensors), a scale on the *X* coordinate axis and the *Y* coordinate axis is a communication hop.

**Figure 4:**
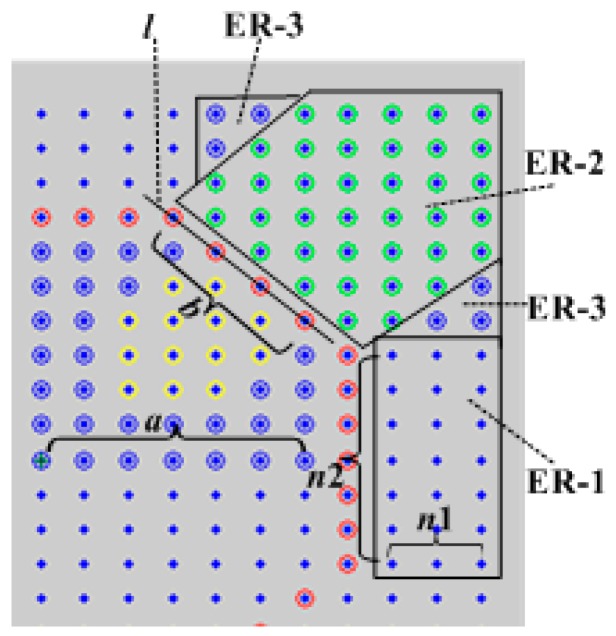
Division of the external area in the center ring (upper right quarter of Figure 3).

In the first place, we discuss the external area ER, which is shown in Figure 4. Assuming each row and each column in network has *n* (even) sensor nodes (because the coordinate starts from 0, and the actual rows and columns are *n* + 1), the distance (number of nodes or communication hops) from center node (*n*/2) in the network to the center ring node in the horizontal (or vertical) direction is *a* (positive integer), and the number of nodes on the center ring bevel (line *l* in the Figure) is *b*.

The minimum value of *a* is 1, and the maximum value is *n*/2. The value of *b* is a random integer from 0 to *a*, so *b* ≤ *a*. In order to calculate conveniently, the external area can be divided into three kinds of situations:

(1) As shown in ER-1 in Figure 4, the number of nodes in the vertical direction of the area is *n*2 = 2(*a* − *b*) + 1, and the number of nodes in the horizontal direction of the area is *n*1=(*n* − 1)/2 − *a*. The distance sum (the hops sum) from nodes in the horizontal direction to center ring nodes is *D*_*ER*-1_ = $\sum_{i=1}^{n1}i$. Therefore, there are four identical ER-1 areas in the network, and the distance sum of this part is 4 × [2(*a* − *b*) + 1] × *D*_*ER*-1_.
(2) As shown in Figure 4, in the area of ER-2 the different nodes are connected into lines which are parallel with the center ring *l* line. It is obvious that the distances from nodes on the line to the nodes on the central ring *l* line separately are 1, $\sqrt{2},\;1+\sqrt{2},\;2\sqrt{2},\;1+2\sqrt{2},\;3\sqrt{2},\;1+3\sqrt{2},$ and so on. There are *n* − 2*a* + *b* lines which are paralleled with *l* in ER-2. Among them from the inside to outwards, the number of nodes on the *i*-th line is recorded as *t*_*i*; and the sum of the value of the *X* coordinate axis (denoted as *x*) and *Y* coordinate axis (denoted as *y*) is *x* + *y* = 2 × (*n*/2 + *a*) − *b* + *i* in each node in the *i*-th line. Then, the shortest distance sum is *D*_*ER*-2_ = $\sum_{i=1}^{n-2a+b}t\_i(\mathrm{mod}(i,2)+\mathrm{fix}(i/2)\times \sqrt{2}$ from all the nodes in this area to the node in the *l* line of center ring. Also, mod(*i*,2) is a function: if *i* is an odd number, its value is 1; if *i* is an even number, its value is 0. fix(*i*/2) indicates an integral part that *i* is divided by 2. There are four identical ER-2 areas in the network, and the distance sum of this part is 4 × *D*_*ER*-2_.
(3) For the ER-3 area in Figure 4, if *a* > *n*/2 − 2, the number of nodes in the ER-3 is 0; the distance sum of this part is *D*_*ER*-3_ = 0. If *a* ≤ *n*/2 − 2, the value of *X* coordinate axis (the node on this axis) is *n*/2 + *a* + 2 ≤ *x* ≤ *n*. The value of *Y* coordinate axis is less than its *X* coordinate axis: it is *n*/2 + *a* − *b* + 1 ≤ *y* ≤ (*x* − *b* − 1). For each node in the *i*-th line (connect nodes into lines that are parallel with *X* and from below to upward), if the value of *X* coordinate axis *x* is subtracted by *n*/2 + *a* + *i*, and is marked as *x*′ = *x* − (*n*/2 + *a* + *i*), where *x*′ is from 1 to max(1,*n*/2 − *a* − 1 − *i*).

In the meantime, for each node in the area, the value of the *Y* coordinate axis is *y*, that it is subtracted by the minimum value of the *X* coordinate axis, with 1 added, and is marked as *y*′ > 1. Hence, the shortest distance from each node in the area to the center ring node is *x*′ + *y*′$\sqrt{2}$, where *x*′ is from 1 to *n*/2 − *a* − 1; *y*′ are from *n*/2 + *a* − *b* + 1 to (*x′* − *b* − 1), and *x′* ≥ *y*′. Connecting nodes into lines that are parallel to the *Y* coordinate axis, there are *n*/2 − *a* − 1 lines orthogonal to the *X* coordinate axis. From inside to outward, with the number of nodes in the *i*-th line recorded as *t*_*i*, the sum of *x* and *y* is min(*n*/2 + *a* + 2,*n*) + *n*/2 + *a* − *b* + 1 + *i* in each node in the *i*-th line. This is because *x* is from *n*/2 + *a* + 2 to *n*, and *y is* from *n*/2 + *a* − *b* + 1 to (*x* − *b* − 1). There are eight identical ER-3 areas in the network, so its distance sum is *D_ER-_*_3_ = 8 × $\sum_{i=1}^{n/2-a-1}t\_i(x^{\prime }+y^{\prime }\sqrt{2})$.

Combined with the above analysis, the distance sum *D_ER_* from the nodes in the external area ER to the center ring node is shown as Equation (2):

(2) $$D_{ER}=4\times [2(a-b)+1]\times D_{ER-1}+4\times D_{ER-2}+8\times \sum_{i=1}^{n/2-a-1}t\_i(x+y\sqrt{2})$$

**Figure 5:**
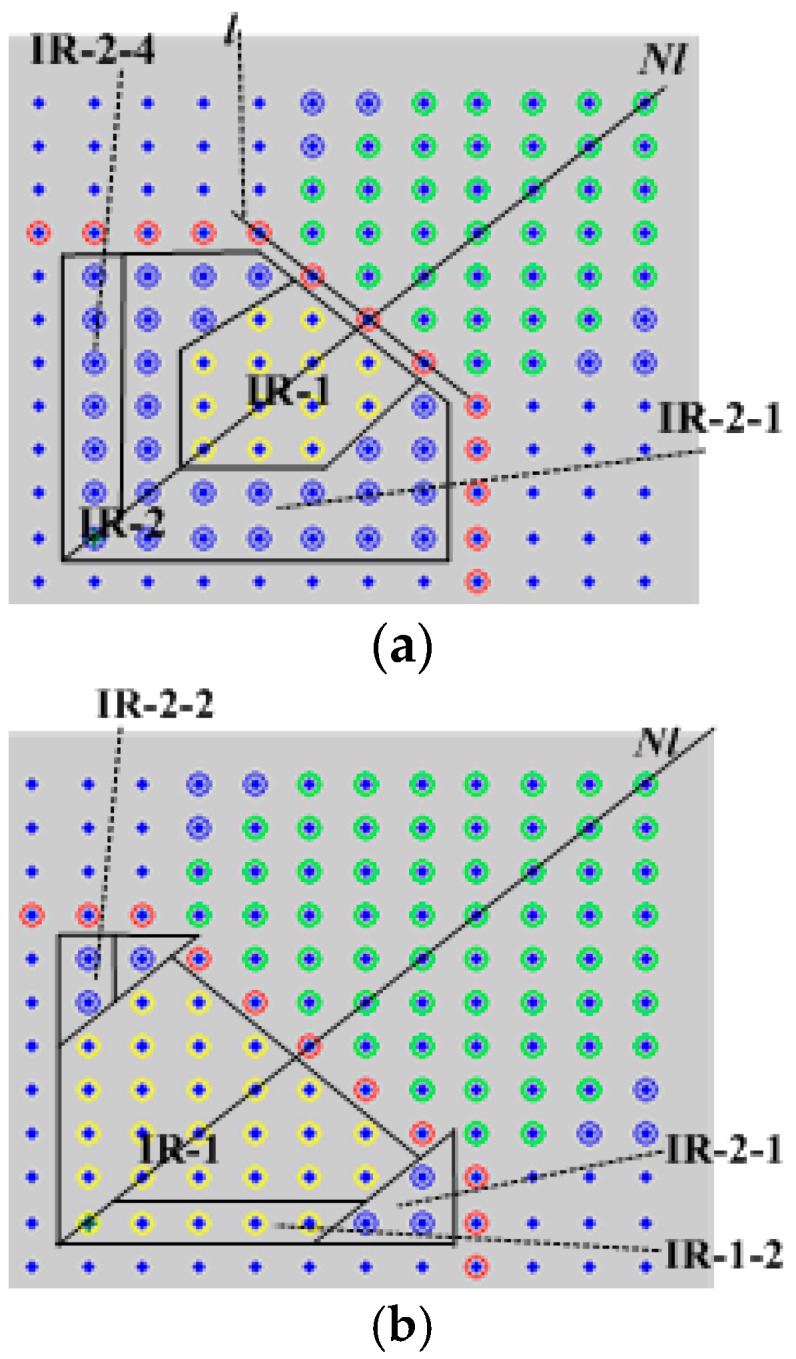
The inside division of the center ring (upper right quarter of Figure 3). (**a**) The center ring does not belong to IR-1; (**b**) The center ring belongs to IR-1.

The internal area (IR) situation of the center ring is discussed below, and is shown in Figure 5. The node in this part meets that sum of the value its *X* coordinate axis and *Y* coordinate axis is *x* + *y* < 2 × (*n*/2 + *a*) − *b*, and *n*/2 ≤ *x*, *y* ≤ *n*/2 + *a* − 1. 

The shortest distance sum from all nodes in IR-1 to the center ring nodes is recorded as *D_IR-_*_1_, and the shortest distance sum from all nodes in IR-2 to the center ring nodes is recorded as *D_IR_*_-2_.

(1) if *a* = 1, there is only one node in IR, and the area IR-1 does not exist. Thus, *D_IR-_*_1_ = 0, *D_IR-_*_2_ = 1.
(2) if *b* ≤ 1, there is no existing IR-1 area in the network, and *D_IR-_*_1_ = 0. As a result, the shortest distance sum from all nodes in area IR-2 to the center ring node is *D_IR-_*_2_ = $\sum_{i=1}^{a-1}i[2\times (2a-(2i-1))+2\times (2a-(2i+1))]$.
(3) if *b* = 2, there is only one node in area IR-1, and then *D_IR-_*_1_ = $\sqrt{2}$.
(4) if *b* > 2, the situation of IR-1 is similar to the ER-2. The distances from nodes on different lines that are parallel *l* line to the center ring nodes are equally similar: $1,\;\sqrt{2},\;1+\sqrt{2},\;2\sqrt{2},\;1+2\sqrt{2},\;3\sqrt{2},\;1+3\sqrt{2}$, and so on. In IR-1, there are *b* + 2 lines that parallel the line *l*. From inside to outside, the number of nodes in the *i*-th line recorded as *t*_*i*, and for the nodes on the *i*-th line, the sum of the value of *X* coordinate axis and *Y* coordinate axis is *x* + *y* = 2 × (*n*/2 + *a* − (*b* + 1)) + *i*, at the same time *x* + *y* < 2 × (*n*/2 + *a* − *b*), *x* is from max(*n*/2, *n*/2 + *a* − (*b* + 1)) to *n*/2 + *a* − 2, and *y* is from max(*i* − *b* + 2, max(*n*/2, *n*/2 + *a* − (*b* + 1))) to min(*i* + *b* − 2, *n*/2 + *a* − 2). Thus, the shortest distance sum from all nodes in IR-1 to the center ring node is *D_IR-_*_1_ = $\sum_{i=1}^{b+2}t\_i(\mathrm{mod}(i,2)+\mathrm{fix}(i/2)\times \sqrt{2})$.

The number of nodes in area IR-2 is obtained when the number of all nodes in IR is subtracted from the number of nodes in IR-1. The distance of the nodes in the opposite side along the horizontal or vertical direction is respectively 1, 2, 3, and so on. Because the size of the IR-1 will affect the size of the IR-2, it is necessary to discuss the distance sum from nodes in IR-2 to the center ring nodes according to the size of the IR-1 region. Figure 5a shows the case where center node does not have a location in IR-1:

① IR-2-1 is below the diagonal line in IR-2, and *D_IR_*_-2-1_ represents the shortest distance sum from the nodes (excluding the nodes on diagonal line) in this area to the center ring nodes. Then, *D_IR_*_-2-1_ = $\sum_{i=1}^{a-1}(a-i)t\_i$. Where the number of nodes in this area are recorded as *t*_*i*, the values of the *X* coordinates axis are *x* = *n*/2 + *i*, and for the *Y* coordinates axis *y* < *n*/2 + *i*.
② The distance from the center node to the center ring is *D_IR_*_-2-2_, with the value *a*.
③ The *n_IR_*_-2-3_ is the number of nodes (excluding the center point) on the diagonal line *Nl.* The shortest distance sum from them to the center ring node is recorded as *D_IR_*_-2-3._ Then, we find that *D_IR_*_-2-3_ = $\sum_{i=1}^{n_{IR-2-3}}\left(a-i\right)$.
④ IR-2-4 represents the area of center node in the vertical (or horizontal) direction (excluding the center point). *D_IR_*_-2-4_ represents the distance sum from those nodes in this area to the center ring nodes. As a result, we can find that *D_IR_*_-2-4_ = $\sum_{i=1}^{a-1}\left(a-i\right)$.

Combined with the above analysis, the distance sum *D_IR_* from the nodes in the internal area to the center ring nodes is shown as Equation (3):

*D_IR_* = 4 × *D_IR-_*_1_ + 4 × [2 × *D*_*IR*-2-1_ + *D*_*IR*-2-3_ − *D*_*IR*-2-4_] + *D_IR_*_-2-2_ (3)

Figure 5b shows the case where IR-1 contains the center node:

① For IR-2-1, the number of nodes in the horizontal and vertical direction is *a* − *b* + 1, and the distance sum is *D_IR_*_-2-1_ = $\sum_{i=1}^{a-b+1}i\left(a-b+2-i\right)$.
② IR-2-2 represents the area of center node in a vertical (or horizontal) direction (excluding the center point) and belongs to the area IR-2. The number of nodes is *a* − *b* + 1, and its distance sum is *D_IR_*_-2-2_ = $\sum_{i=1}^{a-b+1}i$.
③ D*_IR_*_-1-1_ indicates the distance from the center node to the center ring node, and then there is *D_IR_*_-1-1_ = mod(*b* + 2,2) + fix((*b* + 2)/2).
④ IR-1-2 represents the area of center node in vertical (or horizontal) direction (excluding the center point) and belongs to the area IR-1. The number of nodes in this area is 2*b* − *a*. The distance sum from those nodes to the center ring node is *D_IR_*_-1-2_ = $\sum_{i=1}^{2b-a-1}\mathrm{mod}(2b-a-1-i,2)+\mathrm{fix}((2b-a-1-i)/2)\times \sqrt{2}$.

Thus, for Figure 5b, the shortest distance sum *D_IR_* from the nodes in the internal area IR to the center ring nodes is shown in Equation (4):

*D_IR_* = 4 × [2 × *D_IR_*_-2-1_ − *D_IR_*_-2-2_ + *D_IR-1_* − *D_IR_*_-1-1_ − *D_IR_*_-1-2_] + *D_IR_*_-1-1_ (4)

For building the center ring, due to observation node in the network being regarded as the base station for deploying the sensor nodes, before building the center ring all the center ring nodes are selected out based on Equations (1)–(4), and their parameters are determined. After deploying the sensor nodes, the base station broadcast packets with the center ring parameters to every node in network. Where a node receives the center ring parameters packets, it will respectively get the hop jump number *h_i_* from the base stations; in the meantime, the number of hop *h_i_* will be set as *h_i_* = *h_i_* + 1, and it will send the packets forward to the outer (or inner) nodes, as well as finding the nearest center ring node. Then, the center ring node sends a broadcast packet within one hop to find neighbor nodes. Where the node receives this broadcast packet, if it is the neighbor node of this center ring node, it will give feedback in a message to build a neighbor relationship. In this process, the routing mechanisms of GPSR are used to establishing contact routing in the clockwise or counterclockwise direction around the center ring.

#### 4.2.2. Diffusion and Synchronization of Metadata

After the metadata is generated by the storage node, it will be diffused and synchronized to the center ring node. In this process, the metadata is sent to the center ring node that is the nearest from it after metadata *MD_i_* is generated in the storage node. The center ring node will compare *MD_i_* with the existing metadata set *MD-Set* that has been stored. If the existing metadata set contains the metadata *MD_i_*, *MD_i_* is discarded; if the existing metadata set does not contain the *MD_i_*, *MD_i_* is incorporated into the existed metadata set and then the metadata *MD_i_* is sent to the next node along both clockwise and counterclockwise directions according to the center ring routing. The same process is performed on the other center ring nodes to complete the diffusion and synchronization of metadata. In the processing of metadata diffusion and synchronization, the center ring node will record the increment of hop number (*Rh_i_* = *Rh_i_* + 1 or *Lh_i_* = *Lh_i_* + 1). If the number of center ring node is *k*, diffusion and synchronization are stopped when *Rh_i_* or *Lh_i_* is equal to ⌈*k*/2⌉. The processing of metadata diffusion and synchronization is described as Algorithm 1:
**Algorithm 1:** Metadata diffusion and synchronization on DAGM   receive mete data *MD_i_* from other sensor nodes;   If (*MD_i_* not exist in *MD-Set*)    *MD_i_* merge into *MD-Set*;    If (not exist *Rh_i_* or *Lh_i_*)     *Rh_i_* = 0, *Lh_i_* = 0;    else if (exist *Rh_i_* or *Lh_i_* and *Rh_i_* > ⌈k/2⌉ or *Lh_i_* > ⌈k/2⌉)     *Rh_i_* = *Rh_i_* + 1 or *Lh_i_* = *Lh_i_* + 1;      send *MD_i_* and *Rh_i_* to right ring sensor node;      or send *MD_i_* and *Lh_i_* to left ring sensor node;   else    delete *MD_i_*;   end if.


#### 4.2.3. The Data Query Sentences Process Mechanism

With each node queries user data from the network, it will send the query sentence to the nearest center ring node from it. Data query sentences include data types, granularity, QoS etc., At first, the query sentence is split into meta-query (*MQ*) sentences in accordance with the metadata where the center ring node receives the data query sentence. Then, the meta-query is compared with the metadata that is stored in this node. If it is found that the data exists in the network, data transmission routing is generated, and the query result “*ready to receive data*” and data transmission routing are returned to the user. In the meantime, the specific data requests and data transmission routing are sent to the data storage node. If there is no data in the network, the query will be ended and the result “*query failed*” is returned to the user. The processing of data query and data transmission routing generation are described as Algorithm 2:
**Algorithm 2:** Data query and data transmission routing generation on DAGM   receive data query *DQ* from other sensor nodes;   decompose *DQ* into meta query *DMQ_i_*;   If (*DMQ_i_* not exist in *MD-Set*)    send *query failed* to user;   else    generate data transmit routing *DTR* based on GPSR;    send *DTR* and *DMQ_i_* to the storage node;    send *DTR* and *ready to receive data* to user;   end if.

### 4.3. Data Storage Strategy

When the sensor nodes generate data, the location of the storage node is determined based on GHT according to the network area partition. According to the system model in the second section, the location of the *k*-th storage node is shown as Equation (5):

(5) $$H(X,\;k)=X_{0}+G_{X}\times g+\frac{g}{k},\;H(Y,\;k)=Y_{0}+G_{Y}\times g+\frac{g}{k}$$

where *G_X_* = $\left\lfloor \frac{X-X_{0}}{g}\right\rfloor$, *G_Y_* = $\left\lfloor \frac{Y-Y_{0}}{g}\right\rfloor$ represent the lattice ID numbers of nodes.

The data transmission routing is calculated out by GPSR routing protocol when the sensor node gets the location of the storage node. Then, the data is transmitted to the storage node. In processing data transmission, each node will send data to the neighbor node that is the nearest from the storage node. When a node’s next hop is itself, this node can be considered a storage node, which stores data locally and generates metadata. The metadata is composed of data content abstract (*CA*) and data storage location abstract (*SA*). The *CA* (*VN*, *VD*, *SN*, *GT*) indicates the data attribute name, attribute values, generation node identification number and generation time, respectively; and *SA* (*GN*, *VN*, *SL*) indicates lattice ID numbers of storage node, data attribute names and storage node identification number, respectively. After that, the metadata is sent to the center ring node that is the nearest from the storage node. When the metadata is transmitted to the center ring node, the transmission is stopped. The data storage process is described as Algorithm 3:

**Algorithm 3:** Data storage on DAGM   sensor node *i* generates data *TD*;   node *i* finds the storage node *k* through GHT;   node *i* generates data transmission routing *DTR* based    on GPSR;   node i sends TD to storage node k based on DTR;   if(*TD* exist in database of node *k*)    insert *TD* into its data table;   else    create data table for *TD* and insert on it;    create metadata *MD_i_*;    send metadata *MD_i_* to ring node nearest *k*;   end if.

### 4.4. User Query Data Mechanism

Due to the different times and different application backgrounds, the requirements for data granularity and QoS are different, so in the user query sentence, the data granularity, QoS, and so on must be specified. When the center ring node receives the query sentence, it will find the metadata set according to the specific requirement. Then, it can obtain specific application data from the corresponding data storage node. Finally, the data will be integrated. The form of integration is a hash map table of multiple values. In the hash map table, a key may correspond to multiple values, and each value is a structure body (including the attribute value, the sensor node identification number, storage time, and so on). According to the specific QoS, the data in hash map table is divided into different level and granularity, and sent to the user according to the requirement. All the data-meeting requirements are sent to the user when the user requires the accurate data. When the user requires the summary data, summary data (such as average, max, min) will be generated and returned to user. The user query process is described as Algorithm 4:
**Algorithm 4:** User query data on DAGM   user *m* generates data query sentences *DQ*;   generate transmission routing *TR* and send *DQ* to    center ring node nearest *k*;   create data receive handle *DRH*;   center ring node processing *DQ* (look at Section 4.1);   if(receive *DTR* and *ready to receive data* from center    ring node)   receive data and insert it to *DRH*;   else if(receive *query failed* from center ring node)    release *DRH*;   end if.

## 5. Performance Analysis of DAGM

The location of the center ring node for metadata will affect the data access performance, as well as the energy consumption. The center ring nodes are selected according to balance and optimization between data query time and the energy consumption of the network. This section discusses the data storage and query time performance, and the network energy consumption.

### 5.1. Data Access Performance Analysis

In the process of data storage, metadata diffusion and data query, the delay includes node processing data time and data packet (DP) transmission delay. The node processing data time mainly includes delay in store-and-forwarding of the data, which is much smaller than the data packet transmission delay. Data packet transmission delay is the sum of the sending time and transmission time. Data transmission delay is mainly determined by transmission bandwidth *b_t_*, so the greater the bandwidth is, the less sending time is spent; transmission time depends on the velocity *v_t_* and distance *r_t_* of the transmission medium in signal carriers. For the data packet in *p* bytes, *m* represents the number of retransmissions, and the transmission delay is *T_DP_* = (*p*/*b_t_*) × (*r_t_*/*v_t_*) × *m*;

Data storage time *T_DStr_* in DAGM consists of three parts. The first part is that the sensor nodes generate data and transmit it to the storage node to complete storing time *T_DTra_.* Another part is that the storage nodes generate metadata and metadata is transmitted to the nearest center ring node time *T_MDTra_*. The last part is the time of metadata completing diffusion and synchronization in the center ring, recorded as *T_MDsyn_*. Hence, we can obtain Equation (6):

*T_DStr_* = *T_DTra_* + *T_MDTra_* + *T_MDsyn_* (6)

In processing the data query, the query sentence is sent to the nearest center ring node. The center ring node will find the metadata set. In the network, there are two possible outcomes: existing data meeting the requirement, and no existing data satisfying the data query. If there is data that meets the query sentence, the center ring node generates data transmission routing according to storage location abstract in the metadata. On one hand, if the query results in “*ready to receive data*” and data transmission routing will be notified to the user; on the other hand, the specific data requests and data transmission routing will be sent to the storage node. After that, the data storage node will transmit the specific application data to the user. If there is no data in the network, the result is returned to user with *failed query*. Therefore, the data query time of no data in the network is *T_DQue_* = *T_SQue_* + *T_RQN_*.

*T_SQue_* represents the interval from user sending query sentence to the center ring node receiving it. *T_RQN_* represents the interval from center ring node sending query result to the user receiving it.

When there is existing data meeting the query in the network, we can obtain Equation (7):

*T_DQue_* = *T_SQue_* + max(*T_RQN_*, *T_SMQ_* + *T_DT_*) (7)

*T_SMQ_* represents the interval from center ring node sending specific data requests and data transmission routing to the data storage node. *T_DT_* represents the interval from the storage node-transmitted specific application data to the user who receives it. Generally, *T_SMQ_* is much less than *T_DT_*.

### 5.2. Energy Consumption Analysis

In UWSN, energy consumption mainly includes the consumption on data processing and consumption on data transmission, as usually the processing energy consumption is much less than the transmission energy consumption. Transmission energy consumption includes data-sending energy consumption (*E_Send_*) and data-receiving energy consumption (*E_Recieve_*). In general, the data-receiving energy consumption is a constant, and data-sending energy consumption has a connection with communication distance, and transmission channel characteristics (bandwidth, error rate, etc.). Thus, the energy consumption is shown in Equation (8):

*E_Tra_* = *E_Send_* + *E_Recieve_* = (*pE_elec_* + (*pE_elec_* + *pf*(*d*))) × *m* (8)

*E_elec_* represents the energy consumed by the circuit. *f*(*d*) is a function of the energy consumption on the transmission data in a specific transmission channel, mainly related to the distance. *m* indicates the time of retransmission. 

Processing energy consumption is mainly the energy consumption of the node *processing* data storage and query sentence, and it is proportional to the query times and amount of data *d* stored on the node, so we can obtain: *E_proc_* = *n_q_* × *e_q_* × *d.*

*n_q_* represents *the* number of storage or query within a unit time, and *e_q_* represents the energy consumption of single query or storage. 

The total energy *consumption* of the network *E_Total_* includes data storage energy consumption *E_DStro_*, metadata diffusion energy consumption *E_MDSyn_*, data query energy consumption (*E_DQue_*) and specific application data transmission energy consumption (*E_DTra_*). Therefore, we can obtain Equation (9):

(9) $$E_{Total}=\sum (E_{DStro}+E_{MDSyn})+\sum \left(E_{DQue}+E_{DTra}\right)$$

That is, the energy consumption of the network is the total energy consumption of all data storage, metadata diffusion and data query in network.

## 6. Experiments and Analysis

### 6.1. Experiment Setup

Performance calculation and comparative analysis of DAGM and GHT are performed through simulated experiments in this section. In the simulated GHT experiments, where users query data, the query sentence is sent to all storage nodes through GHT routing in the network. The query result is notified and application data are compressed in a package that returns to the user along same routing. With regard to the experimental context where the network coverage is in an 80 km × 80 km ocean area, the senor nodes are uniformly deployed. Underwater communication relies on acoustic communication, and the speed of sound velocity in the water is *V* = 1500 m/s, where it is assumed that in the transmission channel, energy consumption of transmission data is cubically proportional in relationship to the distance. Here, two scenarios and three states are set according to different situations on application data and network connectivity. In Scenario 1, the network has application data that meets the query sentence; in Scenario 2, the network does not have application data that meets the query sentence. The differences in sensor node parameter and network connectivity (the number of the network connectivity paths between any two nodes) have a heavy influence on data transmission routing and transmission delay. Therefore, this section sets the following three network states according to the differences in sensor node parameter and network connectivity:

State 1: The sensor node communication radius is *r_b_* = 10 km, the number of deployed sensor nodes is *n_b_* = 400, and average connectivity is *k_b_* = 8.5.
State 2: The sensor node communication radius is *r_b_* = 20 km, the number of deployed sensor nodes is *n_b_* = 100, and average connectivity is *k_b_* = 3.5.
State 3: The sensor node communication radius is *r* = 10 km, the number of deployed sensor nodes is *n_b_* = 400, and average connectivity is *k_b_* = 3.5.

### 6.2. Experiment Results and Comparative Analysis

**Figure 6:**
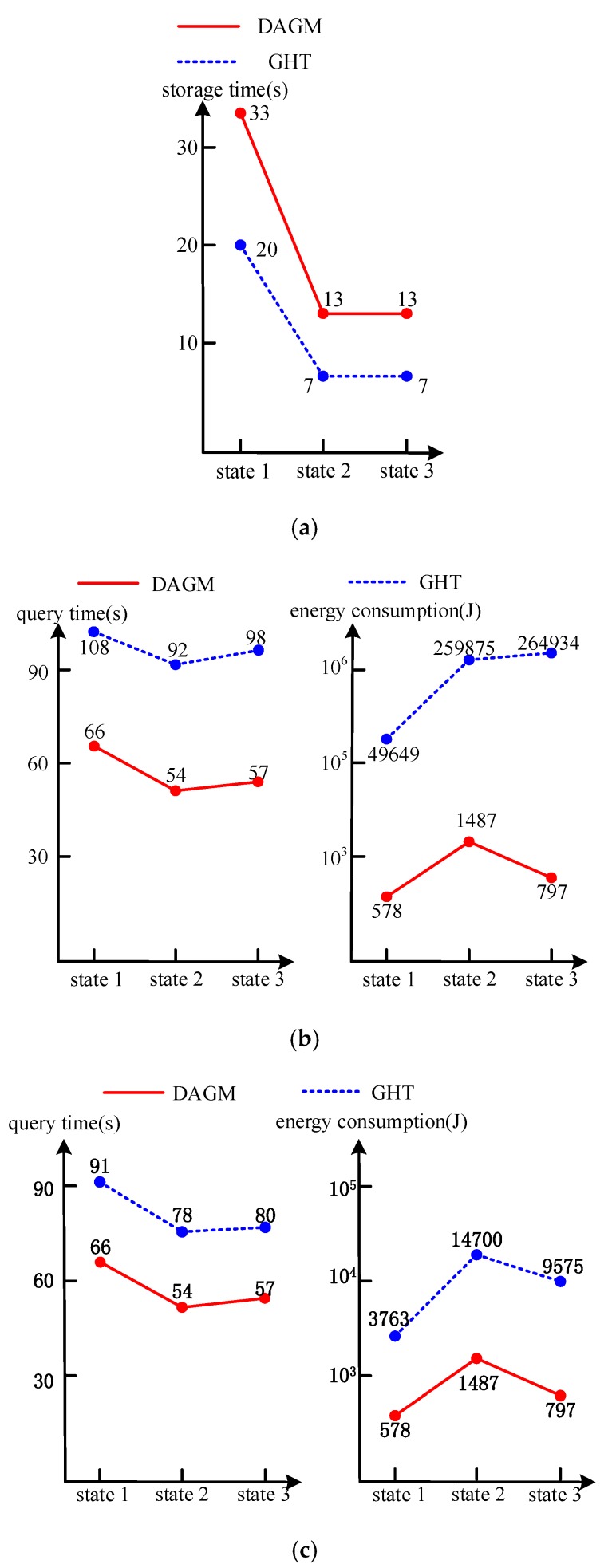
The experiment results where application data exists in the network. (**a**) The performance in storage. DAGM: data access based on a guide map; GHT: geographic harsh tables; (**b**) The performance in global query; (**c**) The performance in partial query.

In the process of the experiment, we assume there are no retransmissions in data storage, metadata diffusion, query data and specific application data transmission. The delay of processing data in the sensor node is less than the delay of the data transmitted, therefore, it is neglected. In the process of data query, there are two kinds of different query methods, which include global query and partial query in the network. In the partial query, one area is randomly selected in the network. In the case of existing application data meeting the requirement, the experiment results of the three methods are shown in Figure 6. In particular, in DAGM the storage time not only includes the time that the sensor node sends the data to the storage node to be stored, but also includes the time where the storage node sends the metadata to the center ring and the diffusion is completed.

From the experimental results, we can draw the following conclusions:

(a) When it comes to storage time performance, in DAGM the senor node generates data and sends it to the storage node. After the storage node stores the data, it also generates the corresponding metadata, and then the metadata is sent to the center ring node and is diffused and synchronized. However, the GHT only saves the data and does not generate metadata; there is no process of diffusing and synchronizing the metadata. Therefore, the time of DAGM in the data storage is more relative than that of GHT (shown in Figure 6a, State 1 is 33 s to 20 s; State 2 and State 3 are both 13 s to 7 s). At the same time, in the global and partial queries, the experimental data storage times have the same results.
(b) In terms of the data query time, this is the interval from the user sending a query sentence to the user receiving specific application data or a query result. In DAGM, the corresponding storage node is purposely located through the data map, so whether it is the global query or a partial query, the storage node will be checked out first. Then, the storage node will send the specific application data through data transmission routing to the user. Thus, the experimental data query times show the same results for a global query and a partial query. However, in GHT, the query sentence is sent to all storage nodes through GHT routing in the network. The specific application data that is required returns along the same routing. Hence, data query delay of DAGM is much better than that of GHT (in global queries, shown in left side of Figure 6b, State 1 is 66 s to 108 s; State 2 is 54 s to 92 s; and State 3 is 57 s to 98 s. In partial query, shown in left side of Figure 6c, State 1 is 66 s to 91 s; State 2 is 54 s to 78 s; and State 3 is 57 s to 80 s).
(c) When it comes to energy consumption, the DAGM sends the query sentence to the nearest center ring node. The center ring node finds the metadata set and then sends specific data requests and data transmission routing to the data storage node with purpose. After that, the storage node will transmit the specific application data to the user through data transmission routing. Data query of GHT directly floods the sentences to all storage nodes in network. The energy consumption is increased exponentially compared with that of DAGM (in global query, shown in right side of Figure 6b, State 1 is 49,649 J to 578 J; State 2 is 259,875 J to 1487 J; State 3 is 264,934 J to 797 J. In the partial query, shown in right side of Figure 6c, State 1 is 3763 J to 578 J; State 2 is 14,700 J to 1487 J; and State 3 is 9575 J to 797 J).
(d) In three different states, the minimal energy consumption of the network is the lowest in state 1 (in DAGM, shown in right side of Figure 6b,c, global query and partial query are 578 J; in GHT, global query is 49,649 J and partial query is 3763 J). This is because the distance between senor nodes is small and there are more connectivity paths between nodes, but the time of data access in State 1 is generally a little more than in states 2 and 3. The best of time performance is in State 2, because of its data transmission with fewer hops. However, because of the large communication distance, energy consumption is also the greatest.

**Figure 7:**
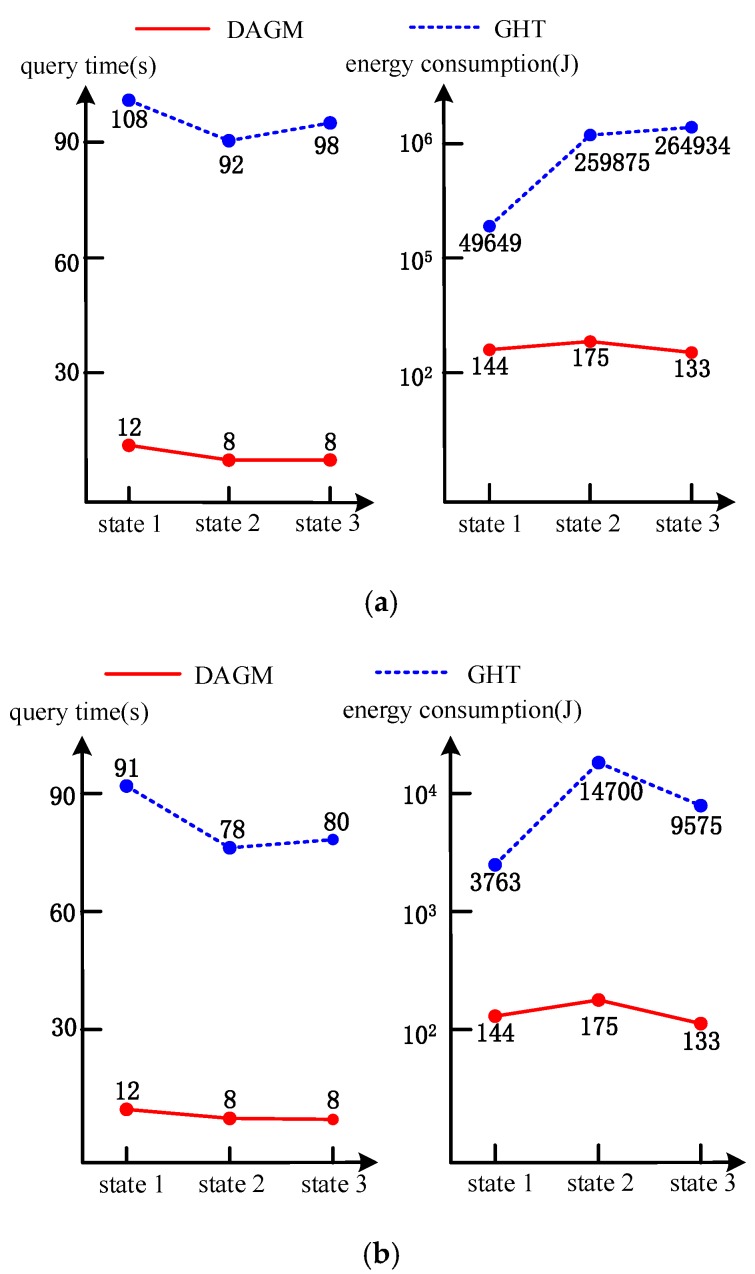
The experimental results where the network does not have application data. (**a**) The performance in global query; (**b**) The performance in partial query.

If there is no application data that meets the query sentence in the network, there is no data storage. In the process of data query, there is also no transmission of the specific application data. The results of the experiment performance are as shown in Figure 7.

From the experimental results, we can draw the following conclusions:

1) When it comes to the query time, the value of performance indexes on GHT do not change compared with Figure 6, because the query data processing is not changed in Scenario 1. In this case, all storage nodes must be checked out, and then the result that the network has no application data will be given. Thus, the query time does not change in GHT. In DAGM, if the center ring node does not find the content abstracts and storage location abstracts of the data that form query sentence through the metadata, it directly returns the result of *query failed*. Therefore, the data query time becomes much shorter (shown on the left side of Figure 7a, from 108 s to 12 s in state 1).
2) When it comes to energy consumption, in DAGM, there are no the routing and specific data requests sent to data storage node, and there are also no specific application data transmitted to the user. Therefore, the energy consumption of the network is of decreased amplitude (in state 1, shown in right side Figure 6b,c and Figure 7, global query and partial query drops from 578 J to 144 J). In GHT, the energy consumption of the network does not decline.
3) In three different states, the minimum change trend of the time performance and total energy consumption is similar in Scenario 1.

**Figure 8:**
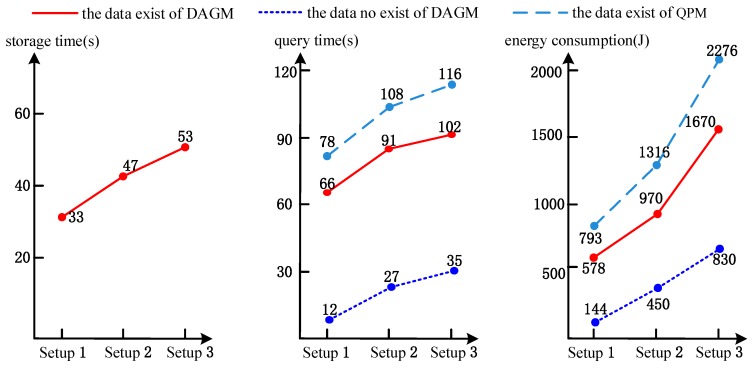
Results of different deployment area size.

After the above experiment, we set the sensor’s communication radius as *r_b_* = 10 km, the number of deployed sensors as *n_b_* = 400, and the average connectivity degree as *k_b_* = 8.5. Based on sensor nodes uniform deployed in 80 km × 80 km (Setup 1) underwater region, we change the network deployment region size, which is respectively 100 km × 100 km (Setup 2) and 120 km × 120 km (Setup 3), and then experiments have been conducted for comparing the performance of our DAGM and QPM^[20]^(2015). In QPM, a routing tree is constructed where a sensor node corresponds to a tree node, and A sensor node acknowledges the fact that whether it lies within the sub-region of interest or not. Sensory data are routed to the sink node leveraging the parent–child relation of sensor nodes specified in the routing tree. So the QPM complements the querying data function which interest in partial, rather than the whole, network region. In the experiment, the routing protocol data and sensory data transmission mechanism were adopted in our technique, and the calculation of energy consumption on data delivery was also adopted in our approach. The experimental results are shown in Figure 8.

As seen in the experimental results shown in Figure 8, when it comes to data storage time and data query time, for DAGM and QPM in Setup 1, Setup 2 and Setup 3, the node deployment in the network just increases the distance between nodes, and then the communication time between nodes increases, so the data storage time and query time increase linearly. However, the energy consumption of the network becomes dramatically larger with the distance between sensor nodes becoming larger, which is mainly due to the cubic relationship between energy consumption and the communication distance. From this perspective, the method of this paper is in line with the general facts. 

Our DAGM provided data storage and query mechanism for UWSNs, at the same time through center ring structure and metadata, data aggregation (mainly large volume non-real time data) and data discovery(include real time and non-real time data and event) can be carried conveniently. Compared with some previous works ([15] (2016), [16] (2016), [19] (2016), [20] (2014), [21] (2015), [31] (2014), [35] (2014), [36] (2015) and [37] (2015)), the functions of the DAGM can be employed as shown in Table 1 (in the Table, √ represents ‘strong support’, -- represents ‘can support’, × represents ‘cannot support’).

From the experimental results, compared with GHT, DAGM has a great advantage when considering from the data access time and energy consumption, although the data storage time is relatively long. Because DAGM query data is directly sent through the metadata to locate storage nodes purposefully, compared with GHT, DAGM achieves a good balance and optimization in energy consumption and data access time.

## 7. Conclusions

With the emphasis on effective safeguarding of national marine rights and interests, the upsurge in marine economic development, and the significant progress in wireless sensor networks, underwater wireless sensor networks (UWSNs) are receiving increasing attention. Underwater acoustic communication is mainly used for UWSNs, which are characterized by low bandwidth, high delay and a higher bit error rates. For specific applications, there is a type of UWSN that can monitor real-time events and application data in the deployment area. It is necessary for the observation node to quickly obtain the events and specific application data it is concerned with in cases such as underwater target monitoring and emergency rescue. It is challenging to store data efficiently generated by sensor nodes, and for specific application data and events to be accessed as quickly as possible by the user while extending the lifetime of the network as much as possible. 

In this paper, the data access based on a guide map (DAGM) method for UWSNs is proposed. In DAGM, metadata is used to describe the data content abstract and storage location abstract. The center ring is based on the shortest average query path of the network, and thus, the metadata is stored in the center ring nodes, then forming a data map of the network. In the process of data query, DAGM chooses the nearest center ring from the user to deal with the query sentence, so that the user can quickly access the specific application data and events that it is concerned with. The results of the simulated experiment show that DAGM provides quicker user access for the specific application data and events, and reduces the total energy consumption of the network while effectively prolonging the lifetime of the network.

In future work, we will mainly focus on how to improve the storage efficiency, decrease the query time and energy consumption of networks under practical underwater conditions. In addition, our approach can support data aggregation and data discovery. Then we will adaptively improve the data map and metadata mechanism on DAGM for them, and provide a data access method with full functions and performances efficiency for UWSNs. Biagi et al. [48] have proposed a new way to look at cognitive access in underwater acoustic communication, and consider it a new research field. We will also improve our approach for this theme in the near future.

## Figures and Tables

**Table 1:** DAGM compared with some other works.

Item	Our Work	Work [15,16]	Work [19,20,35]	Work [21,37]	Work [31,36]
Data storage	√	√	×	×	×
Data aggregation	--	√	√	--	--
Data query	√	--	×	√	×
Data discovery	--	×	√	--	√
